# Association of gestational hypertriglyceridemia, diabetes with serum ferritin levels in early pregnancy: a retrospective cohort study

**DOI:** 10.3389/fendo.2023.1067655

**Published:** 2023-07-19

**Authors:** ZhuYuan Zhang, Xing Li, XueXin Zhou, Yan Zhang, XuPei Gan, XianMing Xu, Hao Wu

**Affiliations:** Department of Obstetrics and Gynecology, Shanghai General Hospital, Shanghai, China

**Keywords:** gestational diabetes, serum ferritin levels, insulin resistance, hypertriglyceridemia, metabolism & endocrinology, gestational diabetes

## Abstract

**Aims:**

Previous studies showed conflicting results linking body iron stores to the risk of gestational diabetes mellitus (GDM) and dyslipidemia. We aim to investigate the relationship between serum ferritin, and the prevalence of GDM, insulin resistance (IR) and hypertriglyceridemia.

**Methods:**

A total of 781 singleton pregnant women of gestation in Shanghai General Hospital took part in the retrospective cohort study conducted. The participants were divided into four groups by quartiles of serum ferritin levels (Q1–4). Binary logistic regressions were used to examine the strength of association between the different traits and the serum ferritin (sFer) quartiles separately, where Q1 (lowest ferritin quartile) was taken as the base reference. One-way ANOVA was adopted to compare the averages of the different variables across Sfer quartiles.

**Results:**

Compared with the lowest serum ferritin quartile (Q1), the ORs for Q3, and Q4 in our population were 1.79 (1.01–2.646), and 2.07 (1.089-2.562) respectively and this trend persisted even after adjusted for age and pre-BMI. Women with higher serum ferritin quartile including Q3 (OR=2.182, 95%CI=1.729-5.527, P=0.003) and Q4(OR=3.137, 95%CI=3.137-8.523, P<0.01)are prone to develop insulin resistance disorders. No significant difference was observed between sFer concentrations and gestational hypertriglyceridemia(GTG) in the comparison among these 4 groups across logistic regressions but TG was found positively correlated with increased ferritin values in the second trimester.

**Conclusions:**

Increased concentrations of plasma ferritin in early pregnancy are significantly and positively associated with insulin resistance and incidence of GDM but not gestational dyslipidemia. Further clinical studies are warranted to determine whether it is necessary to encourage pregnant women to take iron supplement as a part of routine antenatal care.

## 1 Introduction

The morbid effects of gestational iron deficiency on both maternal and fetal outcomes remains a global health problem affecting 10–90% of pregnant women (1) as iron is a detrimental supplement. According to the World Health Organization, daily oral iron supplementation (30-60 mg of elemental iron daily intake) should be a part of routine antenatal care with a purpose of avoiding poor maternal-fetal outcomes including intrauterine growth restriction, premature delivery, and neonatal and perinatal death (1) (2). However, when pregnant women sunk in excessive amounts of iron, it is prone to cause potential damage to newborns and mothers since emerging researches suggested that exposures to high iron during hematopoiesis in early life might induce anemia with profound developmental effects and probably worse erythropoietin sensitivity to limit erythropoiesis (3) (4) (5).

Serum ferritin is a major iron storage protein, a widely used marker for total body Fe stores, with a Nano-sized core of hydrated iron oxide and a cage-shaped protein shell, containing 20% iron. Recently increasing studies have discovered the phenomenon that higher serum ferritin concentrations are also related with metabolic disorders of pregnancy such as gestational diabetes (GDM), serum dyslipidemia, insulin resistance (IR) calculated by indexes such as homeostasis model assessment-insulin resistance (HOMA-IR), homeostasis model assessment-insulin secretion (HOMA-IS), and homeostasis model assessment- β cell function (HOMA-β) (6) (7) (8) (9). On the contrary, there are still other conflicting researches pronounced that iron supplementation does not increase the risk of GDM but benefit a lot for mothers and fetus in terms of pregnancy outcomes (10) (11).

Considering the paucity of researches but with conflicting findings, evaluating the relationship between serum ferritin and metabolic disorders in the Chinese pregnant population, we utilized epidemiological data of pregnant women from Shanghai General Hospital, China to explore the association between serum ferritin levels and the prevalence of metabolic disorders including GDM, serum dyslipidemia, and IR.

## 2 Materials and methods

### 2.1 Study population

This retrospective study used data from department of obstetrics in Shanghai General Hospital on 781 singleton pregnant women aged 21-44 years hospitalized for delivery during January 2021 to Oct 2021 after approval of Ethical Review Board Committee of our institution. Informed patient consent was not required as this was a retrospective research, and this study was conducted in accordance with the Declaration of Helsinki.

Inclusion criteria of the study population were listed as:1)live-birth singleton pregnancy; 2)women’s age ≥18 years old; 3)had their first antenatal care visit between 10 and 20 weeks of gestation. Patients were excluded if they were complicated by 1) stillbirths 2) malformation congenital of the fetus 3)chronic diseases including hypertension, lupus, dyslipidemia, diabetes mellitus, acute or chronic liver diseases through medical history taking at the beginning of enrolment 4) multiple gestations, 5) incomplete medical records.

Among 800 women who met these criteria, 5 refused to participate, 6 had abortions before 24 weeks of gestation, and 4 did not undergo an assessment for GDM diagnosis and 4 for not performing serum lipids tests. Ultimately a total of 781 pregnant women were included in this analysis.

### 2.2 Clinical measurements

All measurements and the sample collections were performed in the morning after an overnight fast. Blood samples was extracted in gestation 10-14 weeks for early pregnancy and 24-28 weeks for middle pregnancy. Pre-pregnancy body mass index (P-BMI) (kg/m (2)) was calculated through weight(kg)/height (2)(m (2)). Enzymatic colorimetric GPO-PAP method (Siemens Healthcare diagnostics Inc) was used to measure serum TGs while serum cholesterol level was calculated by the enzymatic endpoint (CHOD-PAP) method on an automatic analyzer (ADVIA Chemistry XPT). Routine immuno-turbidimetry methods on an automatic analyzer (ADVIA Chemistry XPT) was adopted to quantify ApoA1 and ApoB. Serum low-density lipoprotein-cholesterol (LDL-C) and Serum high-density lipoprotein-cholesterol (HDL-C) levels were directly measured through homogeneous enzymatic colorimetric assay (Siemens Healthcare diagnostics Inc) on an automatic analyzer (ADVIA Chemistry XPT). The quantitative determination of glycosylated hemoglobin (HbA1c) was observed with cyanmethemoglobin. Serum glucose level including 0h, 1h and 2h glucose level was determined by glucose oxidase method. Direct chemiluminescence technology with bright spot sandwich method was used to measure insulin level by a fully automatic biochemical analyzer. HOMA-β HOME-IS and HOMA-IR were calculated using the following formulas. HOMA-β was calculated through the formula: HOMA-β= (20 × insulin)/(glucose − 23·5). HOMA-IR was calculated by the standard formula: HOMA-IR= (glucose × insulin)/22·5. HOME– IS was evaluated using the formula: HOME–IS=1/(FINS×FPG). Insulin resistance is defined as the value of HOMA-IR above or equal 2.69 according to a published paper (12). OGTT was performed in the gestation age between 24 and 28 weeks. GDM was diagnosed if one of the points exceeded the below values: 5.1mmol/L (FBG), 10mmol/l (1h), and 8.5mmol/L(2h). There has not been a consensus about gestational hypertriglyceridemia. We regarded those with TG ≥ 3.4mmol/L(reference cut-off for general population is 1.7mmol/L) as patients diagnosed with gestational hypertriglyceridemia since there is a growing trend of TG in this period.

### 2.3 Statistical analysis

The participants were stratified by sFer levels. One-way ANOVA was adopted to compare the averages of the different variables across sFer quartiles. Chi-square tests were used to analyze statistical differences among the study participant’s characteristics in relation to serum ferritin quartile (Q1-4) groups. Binary logistic regressions were used to examine the strength of association between the different traits and the sFer quartiles separately, where Q1 (lowest ferritin quartile) was taken as the base reference.

## 3 Results

### 3.1 Characteristics of the study population according to the ferritin tertials of early gestation

The baseline characteristics of the 781 subjects and metabolism values are detailed in **Table 1**. To assess the relationship between ferritin levels and abnormal plasma glucose in pregnant women, we compared HbA1c and FBG among these 4 groups. The mean HbA1c of Q4 (5.15 ± 0.35) was significantly higher than the other groups(5.14 ± 0.38, 5.10 ± 0.33, 5.11 ± 0.34). Likewise, it can be observed that the mean value of FBG of Q4 (4.90 ± 0.55)was statistically significantly higher than Q1 (4.75 ± 0.48), Q2 (4.72 ± 0.42) and Q3 (4.79 ± 0.44). However, we didn’t notice any obvious correlations between lipid profiles including TC, TG, H-LDL, L-LDL, ApoA1, ApoB, ApoE and Lp(a ), and values of ferritin in early pregnancy.

**Table 1:** Characteristics of the study population & relationship between ferritin of early pregnancy and metabolism in early pregnancy.

Items/Groups	Q1 (196)	Q2 (195)	Q3 (195)	Q4 (195)	All	*p*
Age	30.61 ± 3.82	29.75 ± 3.98	29.47 ± 4.09	29.31 ± 4.18	29.79 ± 4.04	0.007*
Height	160.80 ± 4.78	160.77 ± 6.70	160.02 ± 4.62	160.29 ± 5.00	160.47 ± 5.34	0.4
Weight	56.53 ± 10.04	56.88 ± 10.33	56.90 ± 10.33	57.03 ± 10.33	56.84 ± 10.33	0.97
PreBMI	21.86 ± 3.78	22.00 ± 3.79	22.22 ± 4.10	22.21 ± 4.12	22.07 ± 3.95	0.778
HbA1c	5.14 ± 0.38	5.10 ± 0.33	5.11 ± 0.34	5.24 ± 0.32	5.15 ± 0.35	<0.01*
FBG	4.75 ± 0.48	4.72 ± 0.42	4.79 ± 0.44	4.90 ± 0.55	4.79 ± 0.48	<0.01*
GA	14.54 ± 1.56	12.00 ± 0.00	12.80 ± 0.29	11.25 ± 0.00	12.99 ± 1.51	0.314
TC	5.28 ± 0.85	5.05 ± 0.87	5.13 ± 1.13	5.14 ± 0.87	5.15 ± 0.94	0.122
TG	1.57 ± 0.59	1.50 ± 0.54	1.75 ± 2.31	1.78 ± 0.73	1.65 ± 1.28	0.097
H-LDL	1.75 ± 0.36	1.68 ± 0.38	1.68 ± 0.37	1.66 ± 0.38	1.69 ± 0.37	0.078
L-LDL	2.55 ± 0.65	2.46 ± 0.70	2.45 ± 0.66	2.49 ± 0.64	2.49 ± 0.66	0.498
ApoA1	1.64 ± 0.22	1.60 ± 0.21	1.62 ± 0.22	1.60 ± 0.22	1.61 ± 0.22	0.181
ApoB	0.78 ± 0.17	0.75 ± 0.19	0.75 ± 0.17	0.77 ± 0.17	0.76 ± 0.18	0.143
ApoE	25.89 ± 10.19	25.19 ± 10.04	29.52 ± 63.96	25.38 ± 9.74	26.50 ± 33.17	0.532
Lp(a)	296.33 ± 385.08	253.10 ± 332.46	230.51 ± 289.11	297.31 ± 418.34	269.28 ± 360.07	0.177
Hb	120.68 ± 15.33	129.01 ± 9.50	130.67 ± 13.83	129.22 ± 14.35	127.39 ± 13.98	<0.01*
Ferritin	13.98 ± 5.78	31.40 ± 5.01	53.58 ± 8.18	116.61 ± 59.33	53.84 ± 49.16	<0.01*
Ferritin	3-23.1	23.2-41	41.1-69.6	69.7-560.6	3-560.6	

Age in years, kg, cm, kg/m2, g, and days are the units of age, weight, height, BMI, review intervals, and mmol/for TC, TG, LDL, HDL, and baby weight, while g/l is for ApoA1 and ApoB.

TC, TG, HDL, LDL, Hb represents Glycosylated hemoglobin, fasting plasma glucose glycated albumin, triglycerides, cholesterol, High density lipoprotein Low density lipoprotein and hemoglobin respectively.

The participants were classified into four groups based on their serum ferritin quartile (Q1: 13.98 ± 5.78μg/L, N=196; Q2: 31.40 ± 5.01μg/L, N= 195; Q3: 53.58 ± 8.18μg/L, N= 195; and Q4: 116.61 ± 59.33μg/L, N= 195) at the beginning of the study, and their characteristics are presented in **Table 1**.

* is displayed when p value is no more than 0.05.

### 3.2 Relationship between ferritin of early gestation and metabolism indexes in middle pregnancy

Association between ferritin and metabolism disorders across the tertials of serum ferritin concentrations was presented in **Table 2**. There were no statistically significant differences in 2-h OGTT level and insulin index, whereas the difference was statistically significant for fasting plasma glucose, 1-h OGTT level, GA, HbA1 c, HOMA-β, HOMA-IS and HOMA- IR, which may be correlated to the incidence of GDM. Unlike lipid levels in early pregnancy, however, TG was found positively correlated with mounting ferritin values in the second trimester.

**Table 2:** Relationship between ferritin of early gestation and metabolism indexes in middle pregnancy.

items/groups	Q1 (196)	Q2 (195)	Q3 (195)	Q4 (195)	All	*p*
HbA1c	5.03 ± 0.36	4.95 ± 0.33	5.42 ± 0.65	5.92 ± 0.78	5.33 ± 0.68	0.00^*^
GA	12.01 ± 1.19	12.12 ± 1.24	11.99 ± 1.17	11.69 ± 0.87	11.9 ± 1.14	0.00^*^
OGTT0	4.46 ± 0.44	4.41 ± 0.43	4.53 ± 0.49	4.56 ± 0.49	4.49 ± 0.47	0.01^*^
OGTT1	7.64 ± 1.78	8.02 ± 1.74	9.74 ± 1.96	10.63 ± 1.81	9.00 ± 2.19	0.00^*^
OGTT2	6.52 ± 1.39	6.56 ± 1.43	6.78 ± 1.51	6.75 ± 1.31	6.65 ± 1.42	0.15
INS0	55.65 ± 30.14	55.76 ± 34.63	63.48 ± 38.50	66.71 ± 37.26	60.41 ± 35.53	0.00^*^
INS1	448.76 ± 290.57	465.60 ± 301.25	493.70 ± 299.65	491.48 ± 308.81	474.88 ± 300.13	0.39
INS2	385.2 ± 268.57	394.75 ± 248.44	419.90 ± 301.01	445.44 ± 296.19	411.32 ± 279.86	0.15
HOMA-β	353.92 ± 365.65	301.24 ± 312.08	221.64 ± 194.50	191.94 ± 116.47	267.24 ± 273.03	0.00^*^
HOMA-IS	0.71 ± 0.22	0.68 ± 0.20	0.55 ± 0.17	0.49 ± 0.11	0.61 ± 0.20	0.00^*^
HOMA-IR	1.53 ± 0.45	1.59 ± 0.45	1.98 ± 0.54	2.15 ± 0.55	1.81 ± 0.56	0.00^*^
TG	2.315 ± 0.76	2.342 ± 0.89	2.539 ± 1.947	2.675 ± 0.968	2.46 ± 1.24	0.01^*^
TC	6.393 ± 1.09	6.207 ± 1.128	38.81 ± 453.85	6.576 ± 1.290	14.56 ± 227.80	0.4
LDL	1.941 ± 0.3772	1.885 ± 0.449	1.887 ± 0.409	1.919 ± 0.447	1.90 ± 0.42	0.44
HDL	3.351 ± 0.8988	3.296 ± 0.921	3.185 ± 0.917	3.249 ± 0.919	3.27 ± 0.91	0.28
ApoA1	1.855 ± 0.22	1.831 ± 0.292	1.856 ± 0.302	1.871 ± 0.252	1.85 ± 0.271	0.49
ApoB	1.004 ± 0.14	0.956 ± 0.291	0.953 ± 0.245	1.183 ± 2.689	1.02 ± 1.358	0.28
ApoE	39.15 ± 31.04	38.81 ± 19.08	40.78 ± 50.77	38.72 ± 17.99	39.3 ± 32.48	0.93
LP (a)	305.74 ± 343.73	241.70 ± 240.96	251.72 ± 308.86	306.27 ± 360.99	276. ± 317.88	0.07
Hb	111.24 ± 14.87	114.62 ± 12.14	115.19 ± 12.77	114.67 ± 12.83	113.92 ± 13.27	0.01^*^
Ferritin	9.52 ± 6.39	10.30 ± 6.22	12.85 ± 8.00	29.93 ± 59.21	15.63 ± 31.26	0.00^*^

* is displayed when p value is no more than 0.05.

### 3.3 The association of serum ferritin in early gestation with GDM and insulin resistance by serum ferritin quartile

To further clarify the correlation between sFer levels and Carbohydrate metabolism, we analyzed Odds ratios and adjusted odds ratios for the association of serum ferritin with insulin resistance by serum ferritin quartile (**Table 3**). Here, IR was measured by HOMA-IR and HOMA-%B of the participants. A positive relation was observed as showed in **Table 2**. Compared with people with Q1, the lowest serum ferritin quartile, we found women with higher serum ferritin quartile including Q3(OR=2.182, 95%CI=1.729-5.527, P=0.003) and Q4(OR=3.137, 95%CI=3.137-8.523, P<0.01)are prone to develop insulin resistance disorders (**Table 4**). This association persisted after adjusting for potential confounders factors.

**Table 3:** Odds ratios and adjusted odds ratios for the association of serum ferritin in early gestation with GDM by serum ferritin quartile (reference group = Q1).

GDM		Q1	Q2	Q3	Q4
model1	*p*	Ref	0.91	0.00^*^	0.00
	OR		0.97	1.79^*^	2.07^*^
	95% CI		0.569-1.652	1.01-2.646	1.089-2.562
model2	*P*	Ref	0.91	0.00^*^	0.00^*^
	OR		0.97	1.80^*^	2.07^*^
	95% CI		0.568-1.65	1.002-2.678	1.998-2.6

Q1-4 were grouped according to serum ferritin quartiles. Q, quartile; OR, odds ratio; CI, confidence interval.

Model 1 shows ORs and their 95% CIs calculated from a logistic regression model. Model 2 shows adjusted ORs and their 95% CIs calculated from a logistic regression model adjusted for age and pre-BMI.

Compared with the lowest serum ferritin quartile (Q1), the ORs for Q3, and Q4 in our population were 1.79 (1.01–2.646), and 2.07 (1.089-2.562) respectively and this trend remained almost same even after adjusted for age and pre-BMI.

* is displayed when p value is no more than 0.05.

**Table 4:** Odds ratios and adjusted odds ratios for the association of serum ferritin in early gestation with insulin resistance by serum ferritin quartile (reference group = Q1).

Insulin resistance		Q1	Q2	Q3	Q4
	*p*		0.519	0.003^*^	0^*^
model1	OR	Ref	1.524	2.182^*^	3.137^*^
	95% CI		0.423-3.486	1.729-5.527	3.137-8.523
	*P*		0.508	0.003^*^	0^*^
model2	OR	Ref	1.542	2.718^*^	3.242^*^
	95% CI		0.428-3.557	1.769-5.985	3.555-9.506

Q1-4 were grouped according to serum ferritin quartiles. Q, quartile; OR, odds ratio; CI, confidence interval.

* Model 1 shows ORs and their 95% CIs calculated from a logistic regression model. Model 2 shows adjusted ORs and their 95% CIs calculated from a logistic regression model adjusted for age and pre-BMI. * is displayed when p value is no more than 0.05.

### 3.4 Association of serum ferritin in early gestation with GTG in pregnancy by serum ferritin

To assess the relationship between ferritin levels and dyslipidemia, OR for the occurrence of hypertriglyceridemia was estimated for participants across the SFer quartiles (**Tables 5**, **6**). However, no significant difference was observed in the comparison among these different groups.

**Table 5:** Odds ratios and adjusted odds ratios for the association of serum ferritin in early gestation with GTG in middle pregnancy by serum ferritin quartile (reference group = Q1).

GTG in middle pregnancy		Q1	Q2	Q3	Q4
model1	*p*	Ref	0.258	0.258	0.056
	OR		0.719	0.719	0.553
	95% CI		0.406	0.406	0.302-1.230
model2	*p*	Ref	0.229	0.298	0.066
	OR		0.703	0.737	0.565
	95% CI		0.396	0.415	0.307-1.233

Q1-4 were grouped according to serum ferritin quartiles. Q, quartile; OR, odds ratio; CI, confidence interval.

* Model 1 shows ORs and their 95% CIs calculated from a logistic regression model. Model 2 shows adjusted ORs and their 95% CIs calculated from a logistic regression model adjusted for age and pre-BMI.

**Table 6:** Odds ratios and adjusted odds ratios for the association of serum ferritin with GTG in early pregnancy by serum ferritin quartile (reference group = Q1).

GTG in early pregnancy		Q1	Q2	Q3	Q4
model1	*P*	Ref	0.243	0.988	0.213
	OR		2.065	1.011	0.247
	95% CI		0.611	0.249	0.027-2.210
model2	*P*	Ref	0.266	0.94	0.216
	OR		2.015	1.057	0.246
	95% CI		0.586-6.925	0.255-4.382	0.027-2.269

Q1-4 were grouped according to serum ferritin quartiles. Q, quartile; OR, odds ratio; CI, confidence interval.

* Model 1 shows ORs and their 95% CIs calculated from a logistic regression model. Model 2 shows adjusted ORs and their 95% CIs calculated from a logistic regression model adjusted for age and pre-BMI.

## 4 Discussion

We investigated the relationship between sFer levels in early pregnancy and metabolic disorders including GDM, insulin resistance, hypertriglyceridemia in early and middle pregnancy, and SCH in early and middle pregnancy. The results of our study revealed that elevated serum ferritin levels are positively independently associated with GDM. Furthermore, an increased β-cell function associated with higher ferritin level was observed in pregnancy period. Nevertheless, no obvious correlation between ferritin levels and hypertriglyceridemia in early and middle pregnancy was found.

Our findings correspond well with the previous study by Cheng et al. (13). Cheng et al. conducted a prospective observational study of 851 Chinese pregnant women, and they concluded that increased serum ferritin concentrations of first trimester are involved with an elevated risk of GDM. A longitudinal study of iron status during pregnancy and the risk of GDM from a prospective multiracial cohort (14) suggested that higher iron stores may be related with the development of GDM from early pregnancy, a >2-fold increased odds of GDM with highest quintiles of ferritin compared with lowest ones, which are also in line with our result. The majority of other previous studies are retrospective researches (15) and they also demonstrated that elevated sFer concentrations is positively related with incidence of GDM. Furthermore, our findings are also in accordance with results from previously published prospective studies in non-pregnant individuals on iron store and type 2 diabetes (16) (17) (18). However, 1 large randomized controlled trial conducted in Hong Kong found no association between iron supplementation and GDM (19). The divergence was potentially owing to various body iron stores, and different dosage and time length of iron supplementation in multitudinous pregnant populations.

In terms of insulin resistance, the findings of our research partially concurs with previous work (20) (21). These studies stated that higher content of ferritin and transferrin at baseline were correlated with HOMA-IR, and low HOMA-%, hyperinsulinemia and the metabolic syndrome anomalies (20).

Based on our current study and previous researches, though WHO suggests daily oral iron supplementation (30-60 mg of elemental iron daily intake) should become a part of routine antenatal care, women with lower serum ferritin levels might benefit from iron supplementation but it is likely to contribute some side effects to those with normal plasma ferritin concentrations. Our research suggests that routine iron supplementation for pregnant women may not that suitable considering risk of GDM. Thus, it is necessary for clinicians to assess iron status of pregnant women in early pregnancy to offer individualized iron supplementation recommendations to reduce the incidence of GDM.

Biologically speaking, the observed positive correlation between sFer and glucose homoeostasis is plausible. The underline mechanism by which elevated fasting SF levels pronounce diabetes, damage beta-cell function and decrease insulin sensitivity have not been fully illuminated. However, it has been noted that heme iron shoulder the responsibility of increasing the body’s iron store and thus leading to oxidative injury to pancreatic cells. Furthermore, growing insulin resistance and elevated insulin secretion from the pancreas could cause pancreatic beta-cell exhaustion (22). Some other researches have revealed that high plasma ferritin mirrors increased iron stores of the body and might be taken as an acute phase inflammatory reactant (23) (24). Besides, increased plasma ferritin levels motivate the inflammatory process inducing elevated insulin resistance,decreased insulin secretion by the pancreas, and hepatic dysfunction (25) Ultimately it follows reduced glucose uptake by muscles and mounting gluconeogenesis, hence spelling the development of GDM (26).

In the year of 2017, Li et al. found that growing serum ferritin levels are significantly related to higher risk of of dyslipidemia, independent of other confounding factors such as age and BMI etc. (8) Nevertheless, our findings did not come in accordance with theirs, since our data showed no statistical significance.

The present study has several advantages. Firstly, we evaluated the relationship between serum ferritin and metabolic disorders in Chinese pregnant population as there is a paucity of studies among Chinese pregnant women. Secondly, we analyzed serum ferritin concentrations in early pregnancy, prior to the diagnosis of GDM, which indicates that plasma ferritin would less be influenced by progress of

GDM. Thirdly, the current study suggests that routine iron supplementation for pregnant women may not that suitable considering risk of GDM, which is innovative. Nevertheless, some limitations are also needed to be noticed:Firstly, this study was conducted in a retrospective cohort with a relatively small sample. Secondly, the information on iron supplement use was not provided, so further investigations on the relation of iron supplement with fetal iron status and GDM are needed. Thirdly, we did not assess concentrations of biomarkers of inflammation, but previous studies indicated that plasma ferritin levels can be slightly influenced by inflammation.

## 5 Conclusions

In summary, findings from our present study suggest that increased concentrations of plasma ferritin in early pregnancy are significantly and positively associated with insulin resistance and incidence of GDM but not gestational dyslipidemia. Similar studies, especially well-designed cohort studies or randomized trials, should be performed in different types of populations, since plasma ferritin concentrations may play an important role for predication and prevention of GDM, IR and their complications. Further clinical studies are warranted to determine whether it is necessary to encourage pregnant women to take iron supplement as a part of antenatal care. In addition, further researches are warranted to clarify the underlying mechanisms. Anyway, in order to prevent excessive iron intake, a lower-dose or intermittent iron supplements instead of regular daily supplementation of iron possibly is more suitable for women without iron deficiency.

## Data availability statement

The original contributions presented in the study are included in the article/supplementary material. Further inquiries can be directed to the corresponding authors.

## Ethics statement

The studies involving human participants were reviewed and approved by Shanghai general hospital. The patients/participants provided their written informed consent to participate in this study. Written informed consent was obtained from the individual(s) for the publication of any potentially identifiable images or data included in this article.

## Author contributions

All the authors participated in designing this study. ZZ and XL collected data. XZ and XG undertook the statistical analyses and interpreted the data. ZZ and YZ wrote the first draft of the manuscript, which was reviewed by all the other authors, especially HW and XX, who also offered further contributions as well as advice.

## References

[1] Drukker L Hants Y Farkash R Ruchlemer R Samueloff A Grisaru-Granovsky S. Iron deficiency anemia at admission for labor and delivery is associated with an increased risk for cesarean section and adverse maternal and neonatal outcomes. Transfusion (2015) 55(12):2799–806. doi: 10.1111/trf.13252 26246160

[2] Auerbach M Georgieff MK. Guidelines for iron deficiency in pregnancy: hope abounds: commentary to accompany: UK guidelines on the management of iron deficiency in pregnancy. Br J haematology (2020) 188(6):814–6. doi: 10.1111/bjh.16220 31568577

[3] Wessling-Resnick M. Excess iron: considerations related to development and early growth. Am J Clin Nutr (2017) 106(Suppl 6):1600s–5s. doi: 10.3945/ajcn.117.155879 PMC5701720 29070548

[4] Zhang Y Lu Y Jin L. Iron metabolism and ferroptosis in physiological and pathological pregnancy. Int J Mol Sci (2022) 23(16). doi: 10.3390/ijms23169395 PMC9409111 36012659

[5] Bowers KA Olsen SF Bao W Halldorsson TI Strøm M Zhang C. Plasma concentrations of ferritin in early pregnancy are associated with risk of gestational diabetes mellitus in women in the Danish national birth cohort. J Nutr (2016) 146(9):1756–61. doi: 10.3945/jn.115.227793 PMC4997275 27511926

[6] Chen L Li Y Zhang F Zhang S Zhou X Ji L. Association of serum ferritin levels with metabolic syndrome and insulin resistance in a Chinese population. J Diabetes its complications (2017) 31(2):364–8. doi: 10.1016/j.jdiacomp.2016.06.018 27426616

[7] Al Akl NS Khalifa O Errafii K Arredouani A. Association of dyslipidemia, diabetes and metabolic syndrome with serum ferritin levels: a middle eastern population-based cross-sectional study. Sci Rep (2021) 11(1):24080. doi: 10.1038/s41598-021-03534-y 34916585 PMC8677797

[8] Li J Bao W Zhang T Zhou Y Yang H Jia H. Independent relationship between serum ferritin levels and dyslipidemia in Chinese adults: a population study. PloS One (2017) 12(12):e0190310. doi: 10.1371/journal.pone.0190310 29272309 PMC5741262

[9] Zimiao C Dongdong L Shuoping C Peng Z Fan Z Rujun C. Correlations between iron status and body composition in patients with type 2 diabetes mellitus. Front Nutr (2022) 9:911860. doi: 10.3389/fnut.2022.911860 35911095 PMC9326402

[10] Kinnunen TI Luoto R Helin A Hemminki E. Supplemental iron intake and the risk of glucose intolerance in pregnancy: re-analysis of a randomised controlled trial in Finland. Maternal Child Nutr (2016) 12(1):74–84. doi: 10.1111/mcn.12139 PMC6860106 24995700

[11] Chan KK Chan BC Lam KF Tam S Lao TT. Iron supplement in pregnancy and development of gestational diabetes–a randomised placebo-controlled trial. BJOG an Int J obstetrics gynaecology (2009) 116(6):789–97. doi: 10.1111/j.1471-0528.2008.02014.x 19432567

[12] Tohidi M Ghasemi A Hadaegh F Derakhshan A Chary A Azizi F. Age- and sex-specific reference values for fasting serum insulin levels and insulin resistance/sensitivity indices in healthy Iranian adults: Tehran lipid and glucose study. Clin Biochem (2014) 47(6):432–8. doi: 10.1016/j.clinbiochem.2014.02.007 24530467

[13] Cheng Y Li T He M Liu J Wu K Liu S. The association of elevated serum ferritin concentration in early pregnancy with gestational diabetes mellitus: a prospective observational study. Eur J Clin Nutr (2020) 74(5):741–8. doi: 10.1038/s41430-019-0542-6 31932742

[14] Rawal S Hinkle SN Bao W Zhu Y Grewal J Albert PS. A longitudinal study of iron status during pregnancy and the risk of gestational diabetes: findings from a prospective, multiracial cohort. Diabetologia (2017) 60(2):249–57. doi: 10.1007/s00125-016-4149-3 PMC6331052 27830277

[15] Chen X Scholl TO Stein TP. Association of elevated serum ferritin levels and the risk of gestational diabetes mellitus in pregnant women: the Camden study. Diabetes Care (2006) 29(5):1077–82. doi: 10.2337/dc06-0164 16644640

[16] Jiang R Manson JE Meigs JB Ma J Rifai N Hu FB. Body iron stores in relation to risk of type 2 diabetes in apparently healthy women. Jama (2004) 291(6):711–7. doi: 10.1001/jama.291.6.711 14871914

[17] Lao TT Chan PL Tam KF. Gestational diabetes mellitus in the last trimester - a feature of maternal iron excess? Diabetic Med J Br Diabetic Assoc (2001) 18(3):218–23. doi: 10.1046/j.1464-5491.2001.00453.x 11318843

[18] Helin A Kinnunen TI Raitanen J Ahonen S Virtanen SM Luoto R. Iron intake, haemoglobin and risk of gestational diabetes: a prospective cohort study. BMJ Open (2012) 2(5):. doi: 10.1136/bmjopen-2012-001730 PMC3467630 23015603

[19] Qiu C Zhang C Gelaye B Enquobahrie DA Frederick IO Williams MA. Gestational diabetes mellitus in relation to maternal dietary heme iron and nonheme iron intake. Diabetes Care (2011) 34(7):1564–9. doi: 10.2337/dc11-0135 PMC3120197 21709295

[20] Vari IS Balkau B Kettaneh A Andreí P Tichet J Fumeron F. Ferritin and transferrin are associated with metabolic syndrome abnormalities and their change over time in a general population: data from an epidemiological study on the insulin resistance syndrome (DESIR). Diabetes Care (2007) 30(7):1795–801. doi: 10.2337/dc06-2312 17416791

[21] Ma H Lin H Hu Y Li X He W Jin X. Serum ferritin levels are associated with insulin resistance in Chinese men and post-menopausal women: the shanghai changfeng study. Br J Nutr (2018) 120(8):863–71. doi: 10.1017/S0007114518002167 30189905

[22] Durrani L Ejaz S Tavares LB Mohyeldin M Abureesh D Boorenie M. Correlation between high serum ferritin level and gestational diabetes: a systematic review. Cureus (2021) 13(10):e18990. doi: 10.7759/cureus.18990 34722008 PMC8545518

[23] Yadav A Saini V Kataria M Jain A. NEED OF IRON SUPPLEMENTATION IN GESTATIONAL DIABETES MELLITUS. Acta endocrinologica (Bucharest Romania 2005) (2017) 13(1):126–8. doi: 10.4183/aeb.2017.126 PMC6525755 31149161

[24] Gautam S Alam F Moin S Noor N Arif SH. Role of ferritin and oxidative stress index in gestational diabetes mellitus. J Diabetes Metab Disord (2021) 20(2):1615–9. doi: 10.1007/s40200-021-00911-2 PMC8630333 34900812

[25] Swaminathan S Fonseca VA Alam MG Shah SV. The role of iron in diabetes and its complications. Diabetes Care (2007) 30(7):1926–33. doi: 10.2337/dc06-2625 17429063

[26] Nah EH Cho S Park H Kim S Cho HI. Associations of complete blood count parameters with pancreatic beta-cell function and insulin resistance in prediabetes and type 2 diabetes mellitus. J Clin Lab Anal (2022) 36(6):e24454. doi: 10.1002/jcla.24454 35561266 PMC9169217

